# *Aureoboletus projectellus* (Fungi, Boletales) – Occurrence data, environmental layers and habitat suitability models for North America and Europe

**DOI:** 10.1016/j.dib.2019.103779

**Published:** 2019-02-23

**Authors:** Łukasz Banasiak, Marcin Pietras, Marta Wrzosek, Alicja Okrasińska, Michał Gorczak, Marta Kolanowska, Julia Pawłowska

**Affiliations:** a Department of Molecular Phylogenetics and Evolution, Institute of Botany, Faculty of Biology, University of Warsaw Biological and Chemical Research Centre, Żwirki i Wigury 101, PL 02-089 Warszawa, Poland; b Department of Plant Taxonomy and Nature Conservation, University of Gdańsk, Wita Stwosza 59, PL 80-308 Gdańsk, Poland; c Institute of Dendrology Polish Academy of Science, Parkowa 5, PL 62-035 Kórnik, Poland; d Department of Geobotany and Plant Ecology, Faculty of Biology and Environmental Protection, University of Lodz, Banacha 12/16, 90-237 Łódź, Poland; e Department of Biodiversity Research, Global Change Research Institute AS CR, Bělidla 4a, 603 00 Brno, Czech Republic

## Abstract

*Aureoboletus projectellus* is a bolete species native to eastern North America, which has been introduced to Central Europe. Here we present summarized data about occurrence of the fungus in both disjunctive ranges based on (1) *de novo* georeferencing of herbarium specimens and occurrence reports; (2) information from peer-reviewed articles, mycological forums and websites; (3) personal observations and (4) from queries sent to Forest Districts and National Parks in Poland. Corresponding background data were acquired from public databases and include range of genus *Pinus* – obligatory mycorrhizal partner of *A. projectellus* – and WorldClim bioclimatic data. Both datasets were fit for purpose of range modelling, i.e. were represented as spatially compatible equal-area raster grids encompassing temperate forest biom in eastern North America and Europe. Additionally, maps of habitat suitability, reflecting association between occurrence and background data, were obtained using maximum entropy approach implemented in MaxEnt.

**Table:** Specifications table

Subject area	*Biology*
More specific subject area	*Biogeography – species range modelling*
Type of data	*Geographic coordinates and maps including definitions of projections and related statistics*
How data was acquired	*Georeferencing of herbarium specimens, surveys, queries to other databases and maximum entropy approach implemented in MaxEnt*
Data format	*Occurrence data are raw, environmental layers and habitat suitability maps were analysed.*
Experimental factors	*not applicable*
Experimental features	*not applicable*
Data source location	*Eastern North America and Europe with emphasis on Central Europe*
Data accessibility	*All the datasets are contained in this article.*
Related research article	*Banasiak Ł., Pietras M., Wrzosek M., Okrasińska A., Gorczak M., Kolanowska M., Pawłowska J. (2019) Aureoboletus projectellus (Fungi, Boletales) - An American bolete rapidly spreading in Europe as a new model species for studying expansion of macrofungi. Fungal Ecology 39: 94-99*

**Table:** 

**Value of the data** • Maps of habitat suitability for *A. projectellus* together with raw occurrence data can be used for efficient monitoring of the fungus dispersal in Europe and studying biology of this potentially invasive species. • Environmental background data for eastern North America and Europe constitute a standardized framework for comparative studies of other ectomycorrhizal fungi including those endangered by the spread of *A. projectellus.* • As *A. projectellus* is a potentially invasive ectomycorrhizal fungus in Europe, it constitutes an excellent model for studying the dynamics of dispersal process as well as for analysing its influence on native ectomycorrhizal communities.

## 1 Data

Raw occurrence data are geographic coordinates of fruiting bodies of *Aureoboletus projectellus* provided in World Geodetic System 1984 (WGS 84) standard. Supplementary Table 1 contains data for the native range – eastern North America, and Supplementary Table 2 – for Europe, where the fungus was introduced. Corresponding background data (range of *Pinus* and bioclimatic variables) and range models are spatially compatible raster data in the ESRI Grid format projected onto a plane using Albers equal-area projection with 25 sq. km cells. Details of projections are provided in either Table 1 or ESRI projection metadata (.prj) files (Supplementary material). Range of the pine is a binary presence/absence grid and for a given data cell, a value was set to presence if at least one pine species was reported from that particular location (Supplementary Grids 1 & 2). Nineteen bioclimatic variables of WorldClim ver. 1.4 were expressed in terms of first five principal components with large water bodies removed (Supplementary Grids 3 & 4). Relative suitability maps, i.e. range models, are in the ‘raw’ output format of MaxEnt (Supplementary Grids 5 & 6) supported by a series of standard diagnostic statistics and plots (Supplementary material).

**Table 1:** Details of Albers equal-area projections used for modeling native and introduced ranges.

	eastern North America (native range)	Europe (introduced)
Spatial extent	30N–55N; 60W–95W	40N–65N; 15W–60E
False easting	10,000,000	−10,000,000
False northing	10,000,000	−10,000,000
Central meridian	77.5W	20E
Standard parallels	35N & 50N	45N & 60N
Latitude of origin	42.5N	52.5N
Linear unit	meter	meter
Datum	WGS 1984	WGS 1984

## 2 Experimental design, materials and methods

### 2.1 Occurrence data

The occurrence data for the native range of *A. projectellus* (Supplementary Table 1) were collected from the Mycology Collections Portal (mycoportal.org), making a query for all the nomenclatural synonyms of the fungus and searching among both preserved specimens and observations of fruiting bodies. All the specimens were georeferenced according to the same standardized protocol using a point radius method [1]. The procedure was based on digitized locality descriptions included in the downloaded database. We used Georeferencing Calculator ver. 20160929 [2] and the GeoLocate batch client [3] as auxiliary tools to make calculations and visualize localities on topographic maps and satellite images.

Sites where *A. projectellus* was reported in Europe (Supplementary Table 2) were taken directly from a recent paper [4] describing the species’ spread, based on information from reviewed articles, mycological forums and websites, personal observations and from answers to queries sent to Forest Districts and National Parks in Poland. Additionally, recent records from Sweden were downloaded from Artportalen (artportalen.se).

### 2.2 Environmental data

We downloaded information about natural distribution of pine species in America from the Geosciences and Environmental Change Science Centre (gec.cr.usgs.gov) where digital representations of tree species range maps are archived from other publications [5], [6]. Data for Europe were collected from a website of the European Forest Genetic Resources Programme (euforgen.org). Information about ranges of pine species was combined and represented as a presence/absence grid projected using Albers equal-area projection with 25 sq. km cells (Table 1 and Supplementary material). For a given data cell, we reported presence if at least one pine species was reported from that particular location (Supplementary Grids 1 & 2).

Nineteen bioclimatic variables were downloaded from WorldClim ver. 1.4 [7] with a resolution of 2.5 arc minutes. First, in order to achieve grids spatially compatible with pine presence/absence data, the angular data grid was projected onto a plane using the Albers projection with the same parameters. Second, to obtain a virtually independent set of variables, PCA was performed using a custom script written in R ver. 3.3.1 [8] and the data were limited to a set of first FIVE principal components that explain more than 95% of observed variance (Table 2). During PCA calculations, American and European datasets were treated as a single artificial continuous region and then separated.

**Table 2:** Loadings of the first five Principal Components of bioclimatic variables.

Bioclimatic variable	Comp. 1	Comp. 2	Comp. 3	Comp. 4	Comp. 5
BIO1 = Annual Mean Temperature	−0.2376	0.3182	0.0803	−0.0904	−0.0255
BIO2 = Mean Diurnal Range (Mean of monthly (max temp - min temp))	−0.0597	0.0453	0.5350	0.0592	0.2153
BIO3 = Isothermality (BIO2/BIO7) (* 100)	−0.2979	0.1188	0.0195	−0.0615	0.1375
BIO4 = Temperature Seasonality (standard deviation *100)	0.2701	−0.0768	0.3377	0.1290	0.0266
BIO5 = Max Temperature of Warmest Month	−0.0695	0.3346	0.3805	0.0271	0.0408
BIO6 = Min Temperature of Coldest Month	−0.2569	0.2629	−0.1833	−0.1039	−0.0698
BIO7 = Temperature Annual Range (BIO5-BIO6)	0.2324	−0.0823	0.4197	0.1267	0.0984
BIO8 = Mean Temperature of Wettest Quarter	0.0335	0.0819	0.2383	−0.5566	−0.6097
BIO9 = Mean Temperature of Driest Quarter	−0.2152	0.2828	−0.0378	0.1263	0.2899
BIO10 = Mean Temperature of Warmest Quarter	−0.1070	0.3483	0.3143	−0.0132	−0.0151
BIO11 = Mean Temperature of Coldest Quarter	−0.2745	0.2591	−0.1010	−0.1074	−0.0313
BIO12 = Annual Precipitation	−0.2822	−0.2458	0.1043	−0.0189	0.0373
BIO13 = Precipitation of Wettest Month	−0.2480	−0.2729	0.0787	−0.2121	0.1776
BIO14 = Precipitation of Driest Month	−0.2856	−0.1923	0.0914	0.2147	−0.1724
BIO15 = Precipitation Seasonality (Coefficient of Variation)	0.1647	−0.0154	−0.0270	−0.5900	0.5596
BIO16 = Precipitation of Wettest Quarter	−0.2465	−0.2810	0.0787	−0.1981	0.1682
BIO17 = Precipitation of Driest Quarter	−0.2936	−0.1881	0.1045	0.1877	−0.1428
BIO18 = Precipitation of Warmest Quarter	−0.1680	−0.3294	0.1650	−0.2558	−0.1453
BIO19 = Precipitation of Coldest Quarter	−0.3064	−0.1358	0.0176	0.1519	0.1192

The available bioclimatic variables include major inland bodies of water, which are obviously not suitable habitats for terrestrial fungus. We erased data for a set of major lakes from bioclimatic layers, including the Great Lakes in the native range of *A. projectellus* and Lake Onega, Lake Ladoga and Lake Vanern in northern Europe (Supplementary Grids 3 & 4).

### 2.3 Models of habitat suitability

Based on the occurrence data of *A. projectellus* from North America, and environmental layers including PCA-transformed bioclimatic variables and distribution of *Pinus* in the region, we used the maximum entropy approach implemented in MaxEnt [9] to construct a model of the relative habitat suitability in the native range.

A preliminary analysis of georeferenced occurrences revealed that they were spatially correlated, i.e. the distribution pattern of the obtained points was strikingly uneven, with clusters consisting of occurrences reported from a single survey of spatial extent limited to few kilometres or consecutive visits to the exactly the same location. Each of the clusters was represented by an artificial point being a result of the following calculations. First, duplicated records having the same coordinates were excluded from the analysis, keeping only a single point for a given location. Next, we performed cluster analysis using the ‘Find Identical’ tool implemented in ArcGIS 10.2 Data Management Tools. Two points were assumed spatially coincident if the distance between them was equal to or less than 5,000 m, and the analysis resulted in formally identified clusters of points. Finally, for each cluster, a geographic mean was calculated using the ‘Mean Center’ tool from the Spatial Statistics extension for ArcGIS 10.2 (Supplementary Table 3).

Bioclimatic data were treated as continuous variables and pine presence-absence records as a categorical (binary) predictor. During each of the 100 replications of the MaxEnt analysis, a different subsample of 75% randomly chosen occurrence records was used to train the model that was finally averaged over the replicates. For each replicate the remaining 25% of occurrences were used to test the model and variability of model parameters between replicates was assessed. We chose the ‘raw’ output format and left all the remaining settings at the defaults (Supplementary Grids 5 & 6). Command line to repeat this species model (Linux-style line breaks):

java density.MaxEnt nowarnings noprefixes -E "" -E Aureoboletus_projectellus ∖
responsecurves jackknife outputformat=raw ∖
outputdirectory=[path to custom output directory] ∖
projectionlayers=[path to Supplementary Grids 2 & 4] ∖
samplesfile=[path to Supplementary Table 3] ∖
environmentallayers=[path to Supplementary Grids 1 & 3] ∖
randomseed noremoveduplicates randomtestpoints=25 replicates=100 replicatetype=subsample ∖
-t Pinus
