# Supplementary material for: Aureoboletus projectellus (Fungi, Boletales) – Occurrence data, environmental layers and habitat suitability models for North America and Europe
Source: Data Brief. 2019 Feb 23;23:103779. doi: 10.1016/j.dib.2019.103779 (PMC6660558; doi:10.1016/j.dib.2019.103779)
Supplement: Supplementary file 1 — Multimedia component 1 [file mmc1.doc]

CONFLICT OF INTREST DECLARATION

We wish to confirm that there are no known conflicts of interest associated with this publication and there has been no financial support for this work that could have influenced its outcome.

We confirm that the manuscript has been read and approved by all named authors and that there are no other persons who satisfied the criteria for authorship but are not listed. We further confirm that the order of authors listed in the manuscript has been approved by all of us. We also understand that the Corresponding Author is the sole contact for the Editorial process and she is responsible for communicating with the other authors about progress, submissions of revisions and final approval of proofs.

Signed by all authors as follows:

*Warsaw, 12.02.2019*

*Łukasz Banasiak,*

*Marcin Pietras,*

*Marta Wrzosek,*

*Alicja Okrasińska,*

*Michał Gorczak,*

*Marta Kolanowska,*

*Julia Pawłowska*
