# Supplementary figures and images for: Aureoboletus projectellus (Fungi, Boletales) – Occurrence data, environmental layers and habitat suitability models for North America and Europe
Source: Data Brief. 2019 Feb 23;23:103779. doi: 10.1016/j.dib.2019.103779 (PMC6660558; doi:10.1016/j.dib.2019.103779)

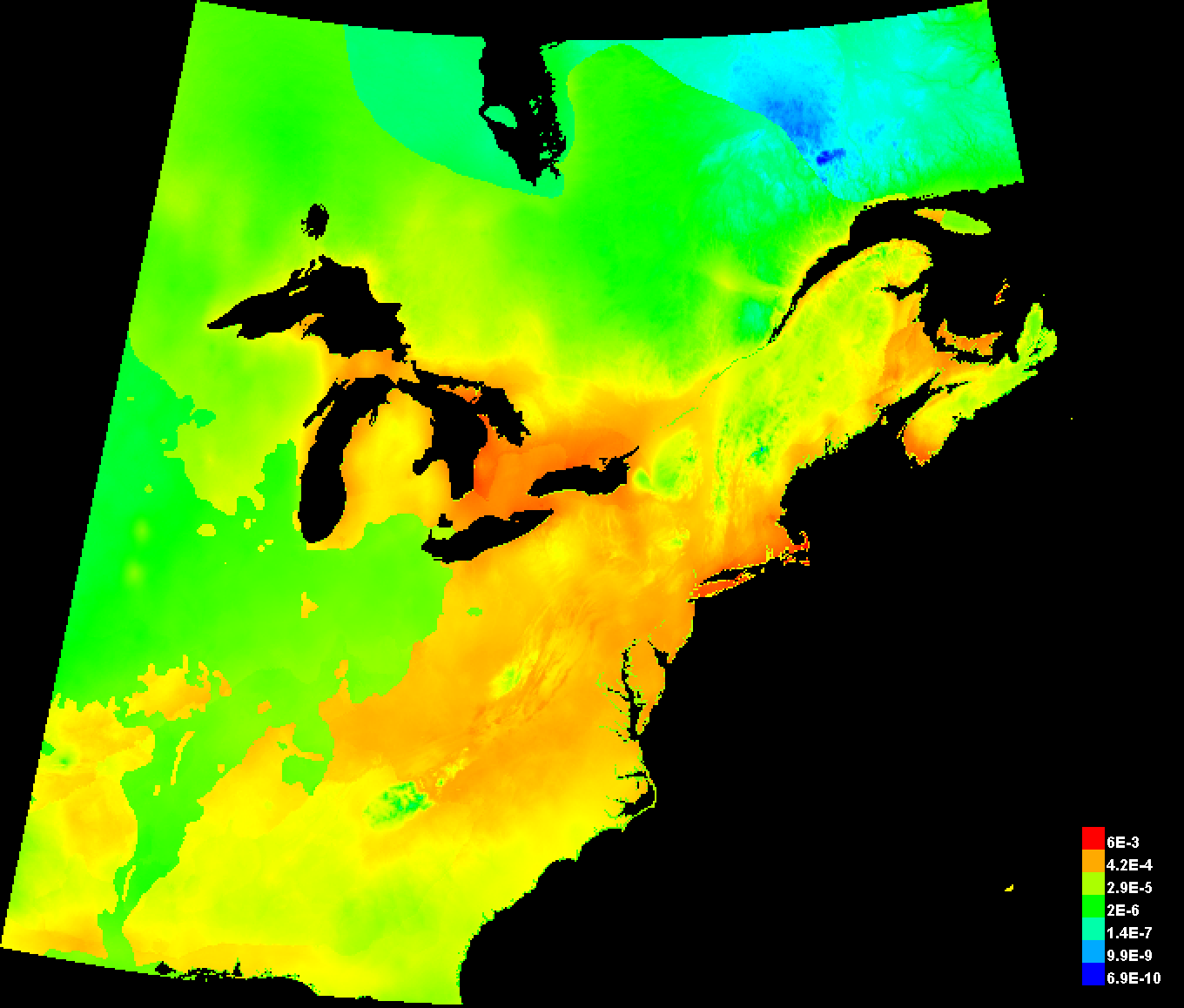

Supplement: Multimedia component 8 [file mmc8.zip › MaxEnt/plots/Aureoboletus_projectellus_avg.png]

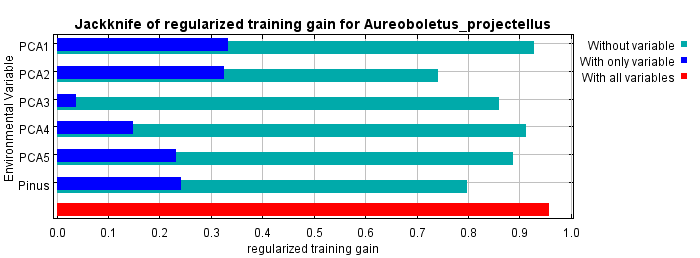

Supplement: Multimedia component 8 [file mmc8.zip › MaxEnt/plots/Aureoboletus_projectellus_jacknife.png]

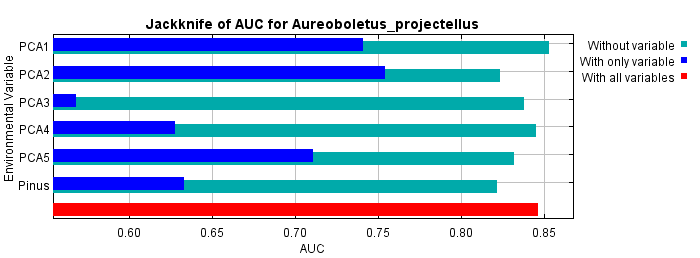

Supplement: Multimedia component 8 [file mmc8.zip › MaxEnt/plots/Aureoboletus_projectellus_jacknife_auc.png]

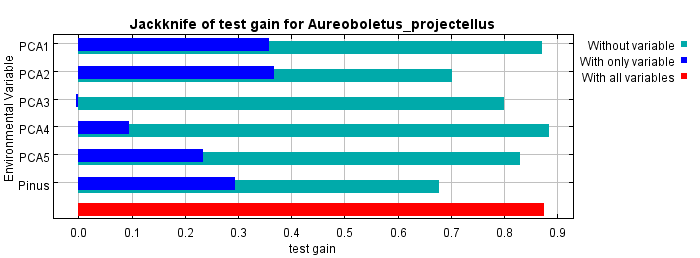

Supplement: Multimedia component 8 [file mmc8.zip › MaxEnt/plots/Aureoboletus_projectellus_jacknife_test.png]

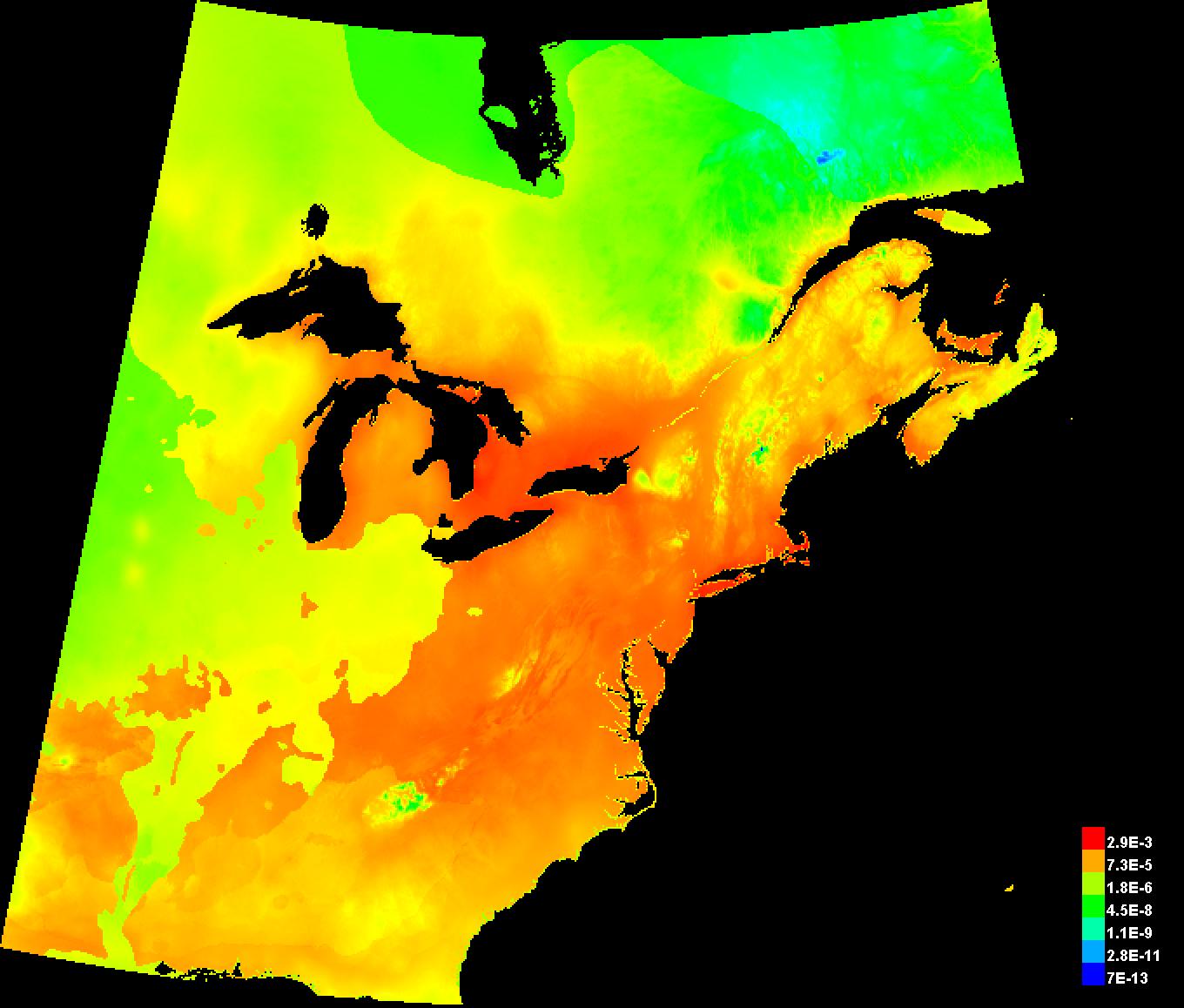

Supplement: Multimedia component 8 [file mmc8.zip › MaxEnt/plots/Aureoboletus_projectellus_lowerci.png]

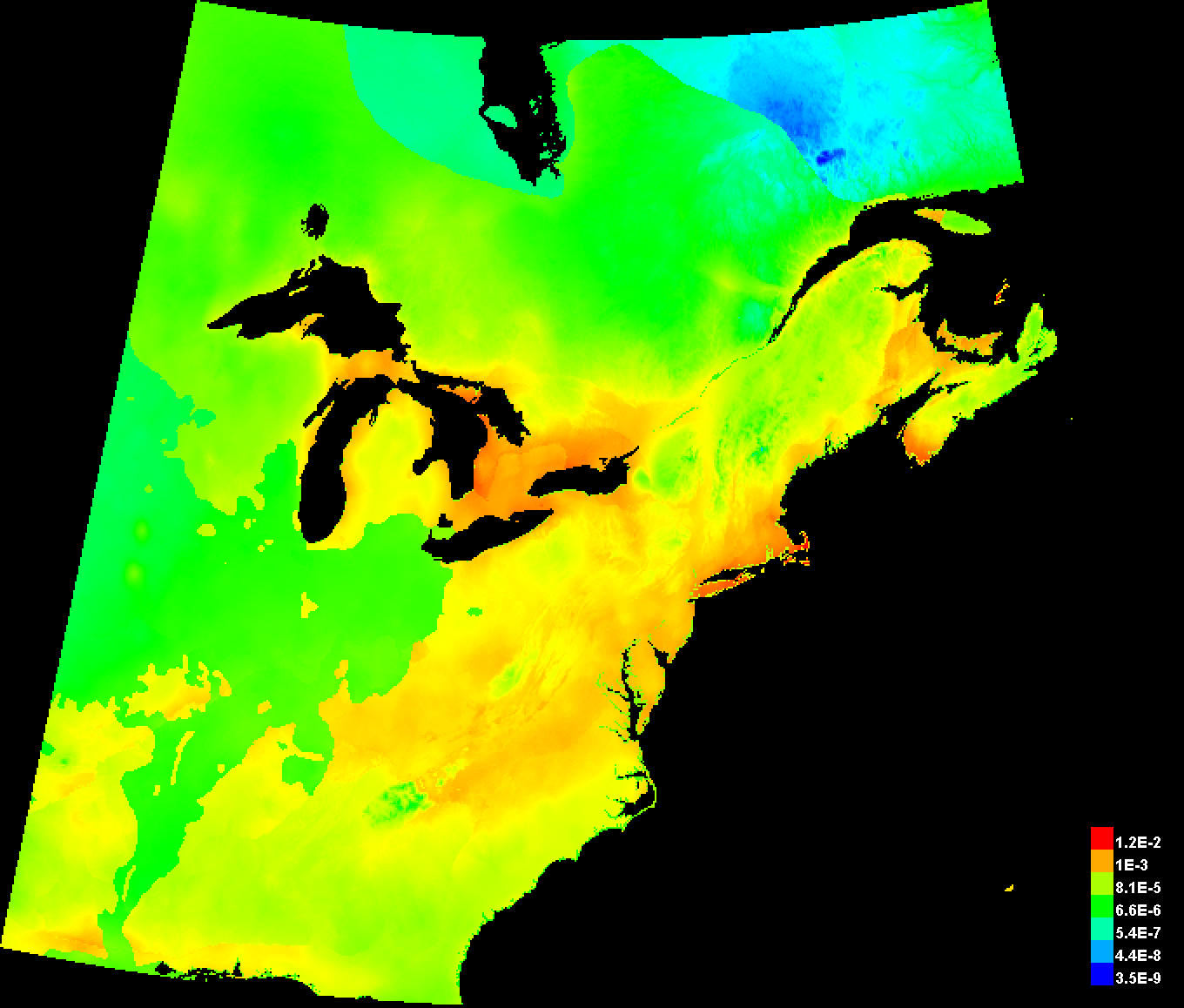

Supplement: Multimedia component 8 [file mmc8.zip › MaxEnt/plots/Aureoboletus_projectellus_max.png]

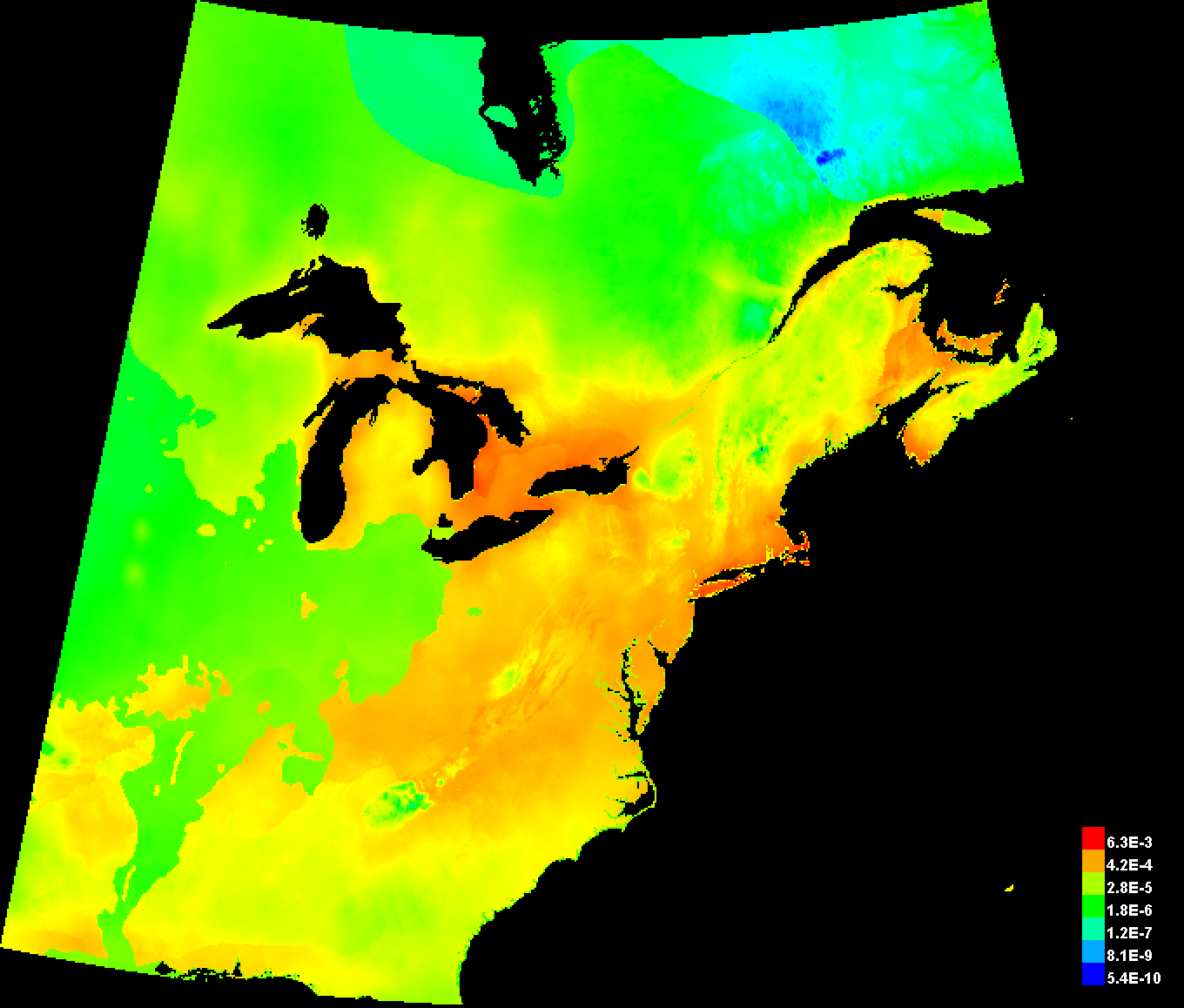

Supplement: Multimedia component 8 [file mmc8.zip › MaxEnt/plots/Aureoboletus_projectellus_median.png]

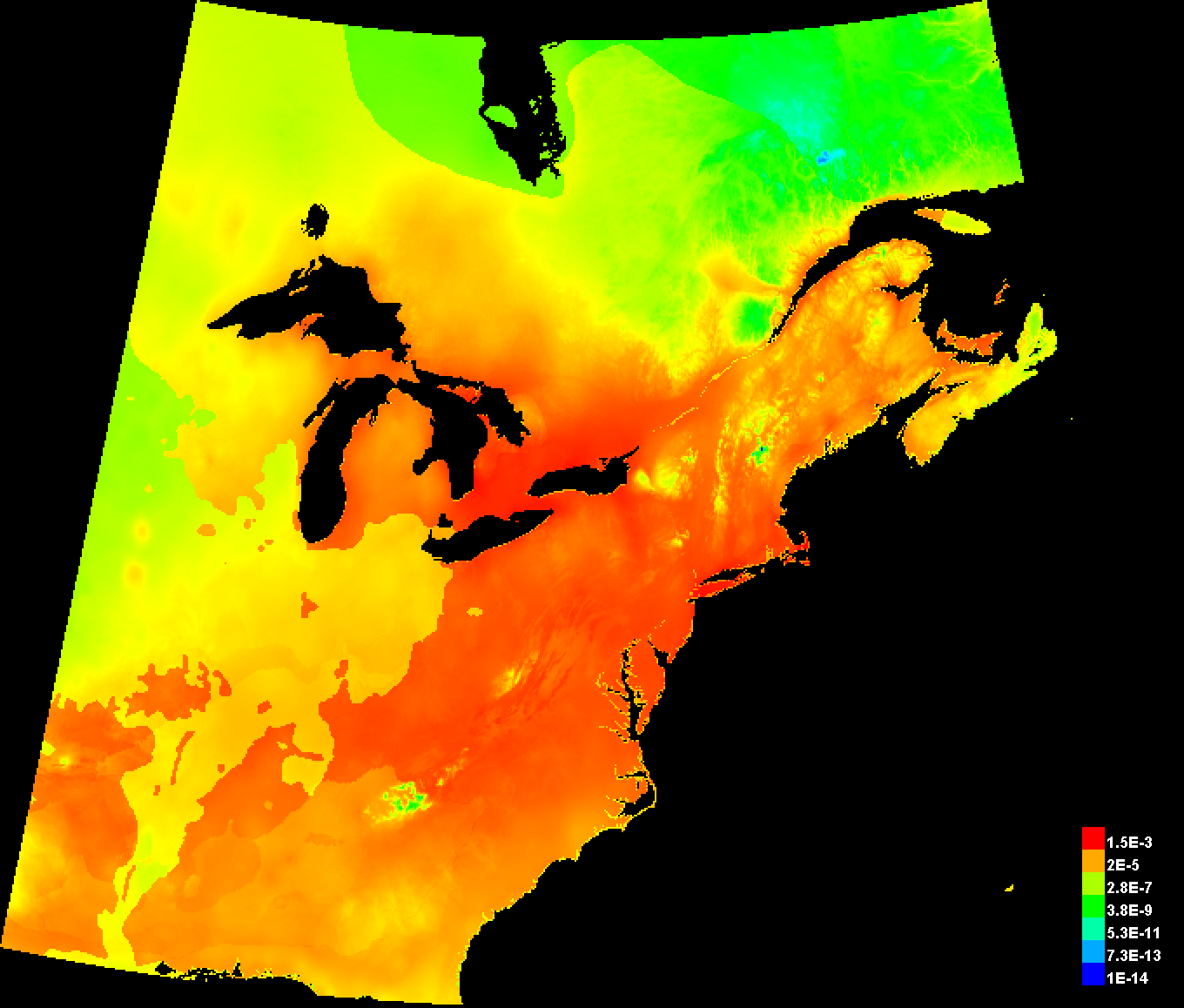

Supplement: Multimedia component 8 [file mmc8.zip › MaxEnt/plots/Aureoboletus_projectellus_min.png]

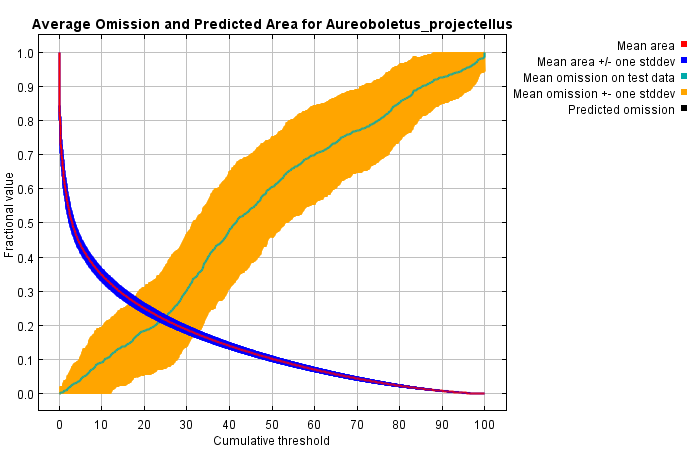

Supplement: Multimedia component 8 [file mmc8.zip › MaxEnt/plots/Aureoboletus_projectellus_omission.png]

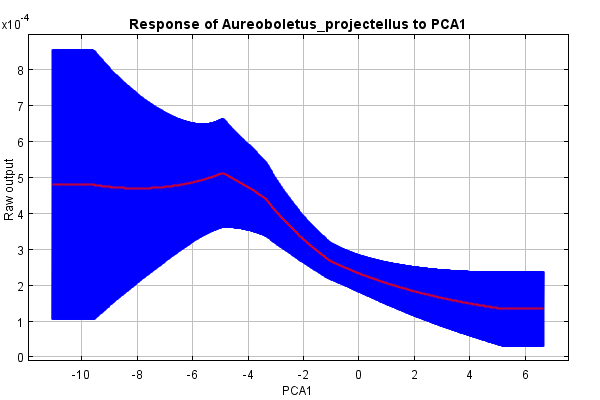

Supplement: Multimedia component 8 [file mmc8.zip › MaxEnt/plots/Aureoboletus_projectellus_PCA1.png]

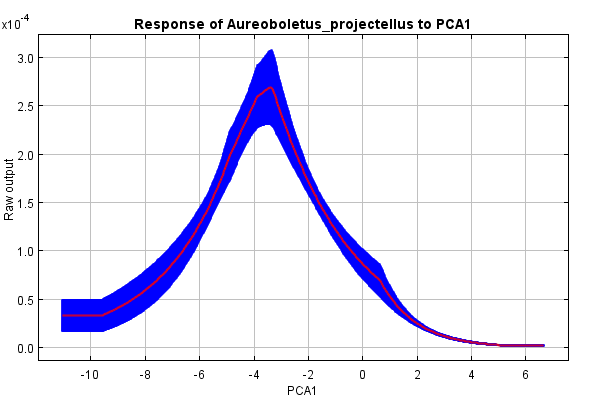

Supplement: Multimedia component 8 [file mmc8.zip › MaxEnt/plots/Aureoboletus_projectellus_PCA1_only.png]

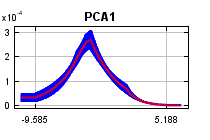

Supplement: Multimedia component 8 [file mmc8.zip › MaxEnt/plots/Aureoboletus_projectellus_PCA1_only_thumb.png]

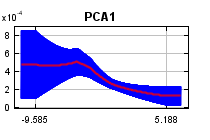

Supplement: Multimedia component 8 [file mmc8.zip › MaxEnt/plots/Aureoboletus_projectellus_PCA1_thumb.png]

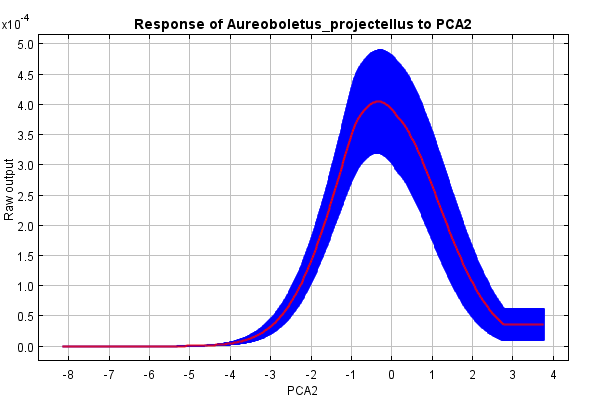

Supplement: Multimedia component 8 [file mmc8.zip › MaxEnt/plots/Aureoboletus_projectellus_PCA2.png]

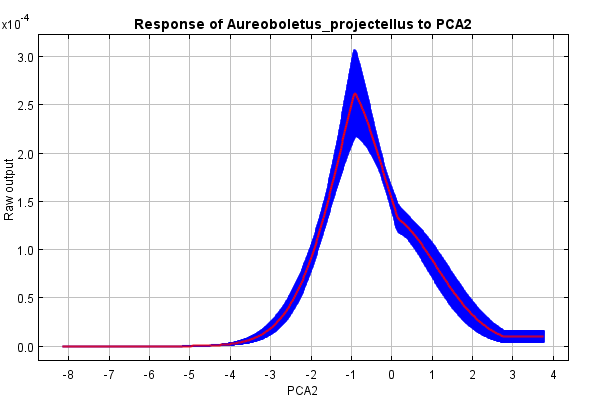

Supplement: Multimedia component 8 [file mmc8.zip › MaxEnt/plots/Aureoboletus_projectellus_PCA2_only.png]

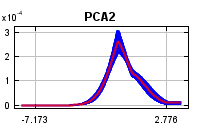

Supplement: Multimedia component 8 [file mmc8.zip › MaxEnt/plots/Aureoboletus_projectellus_PCA2_only_thumb.png]

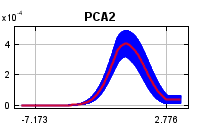

Supplement: Multimedia component 8 [file mmc8.zip › MaxEnt/plots/Aureoboletus_projectellus_PCA2_thumb.png]

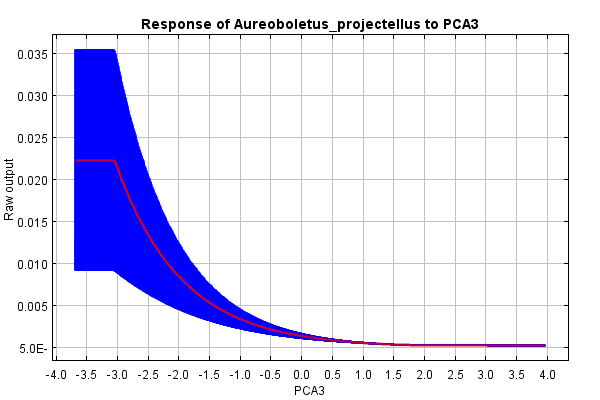

Supplement: Multimedia component 8 [file mmc8.zip › MaxEnt/plots/Aureoboletus_projectellus_PCA3.png]

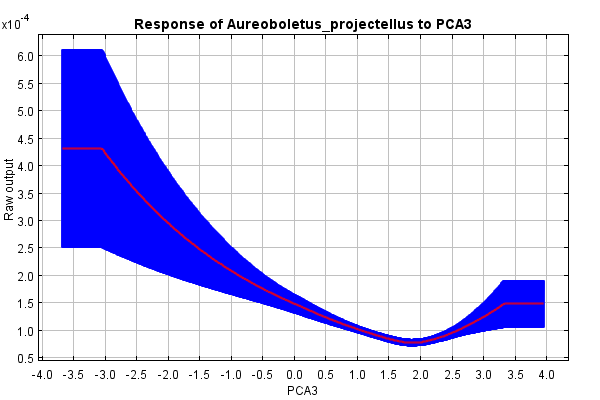

Supplement: Multimedia component 8 [file mmc8.zip › MaxEnt/plots/Aureoboletus_projectellus_PCA3_only.png]

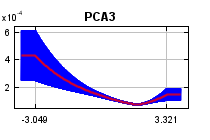

Supplement: Multimedia component 8 [file mmc8.zip › MaxEnt/plots/Aureoboletus_projectellus_PCA3_only_thumb.png]

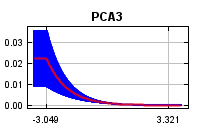

Supplement: Multimedia component 8 [file mmc8.zip › MaxEnt/plots/Aureoboletus_projectellus_PCA3_thumb.png]

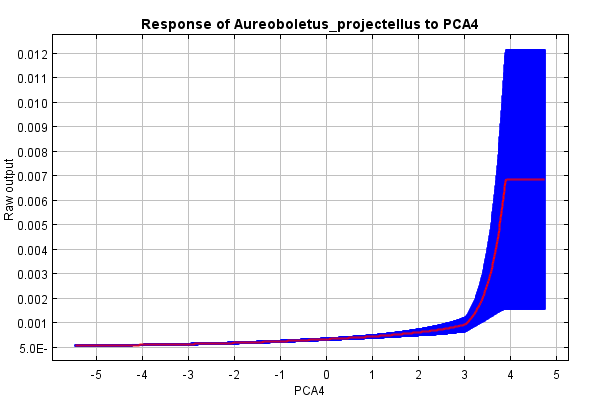

Supplement: Multimedia component 8 [file mmc8.zip › MaxEnt/plots/Aureoboletus_projectellus_PCA4.png]

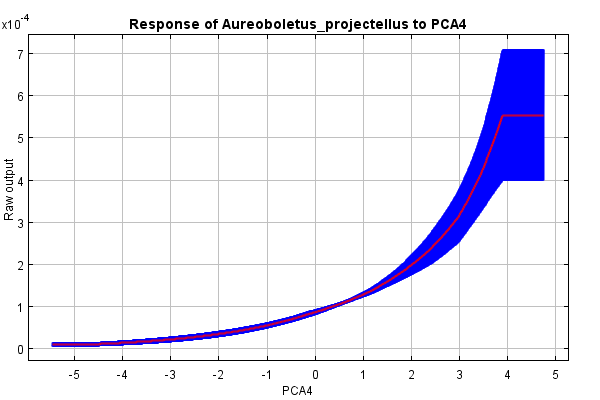

Supplement: Multimedia component 8 [file mmc8.zip › MaxEnt/plots/Aureoboletus_projectellus_PCA4_only.png]

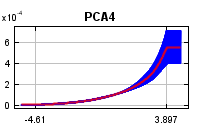

Supplement: Multimedia component 8 [file mmc8.zip › MaxEnt/plots/Aureoboletus_projectellus_PCA4_only_thumb.png]

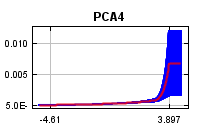

Supplement: Multimedia component 8 [file mmc8.zip › MaxEnt/plots/Aureoboletus_projectellus_PCA4_thumb.png]

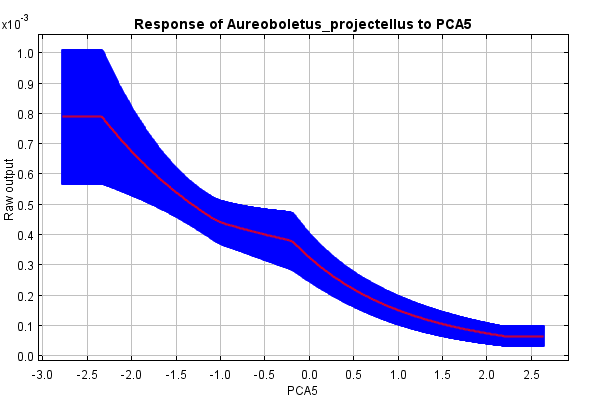

Supplement: Multimedia component 8 [file mmc8.zip › MaxEnt/plots/Aureoboletus_projectellus_PCA5.png]

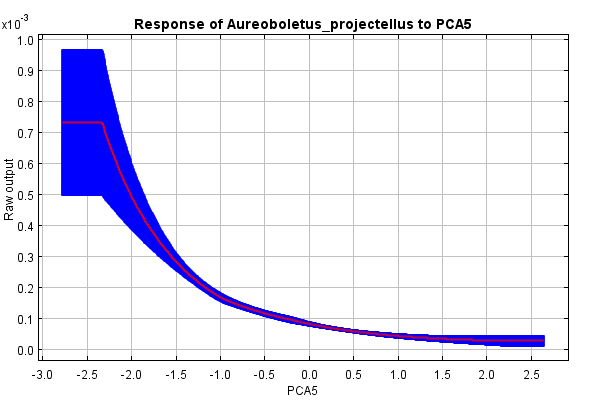

Supplement: Multimedia component 8 [file mmc8.zip › MaxEnt/plots/Aureoboletus_projectellus_PCA5_only.png]

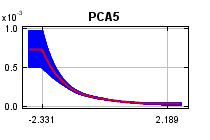

Supplement: Multimedia component 8 [file mmc8.zip › MaxEnt/plots/Aureoboletus_projectellus_PCA5_only_thumb.png]

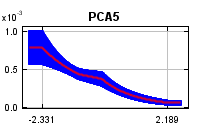

Supplement: Multimedia component 8 [file mmc8.zip › MaxEnt/plots/Aureoboletus_projectellus_PCA5_thumb.png]

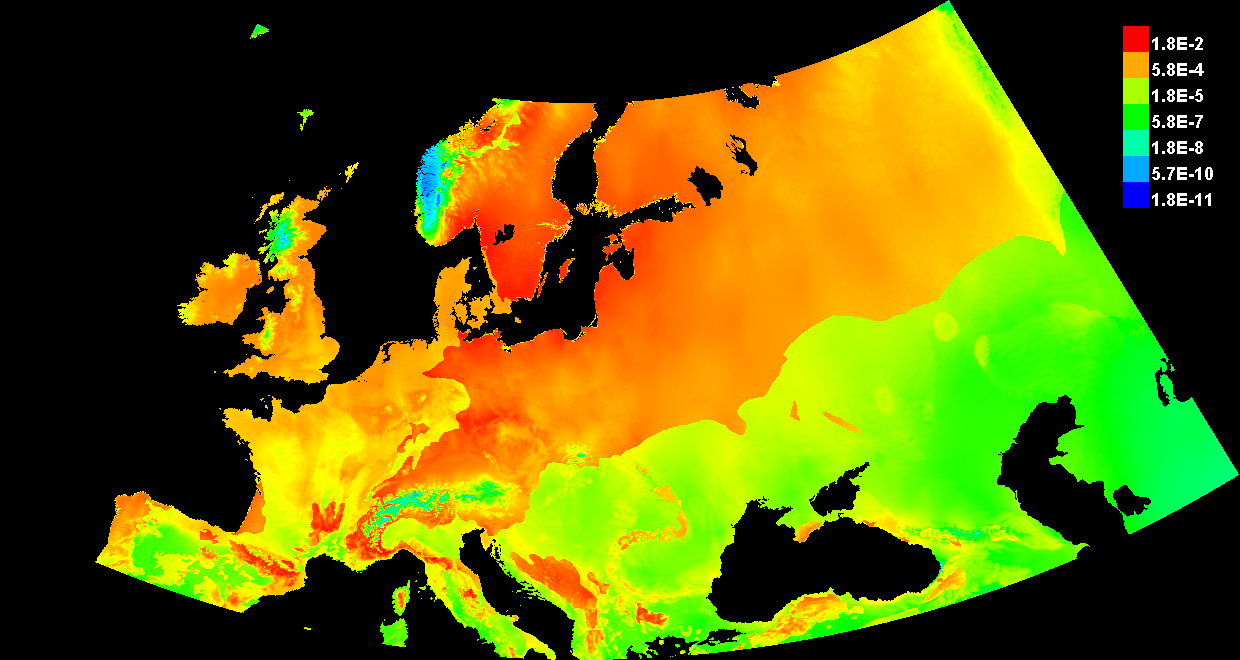

Supplement: Multimedia component 8 [file mmc8.zip › MaxEnt/plots/Aureoboletus_projectellus_PCA_avg.png]

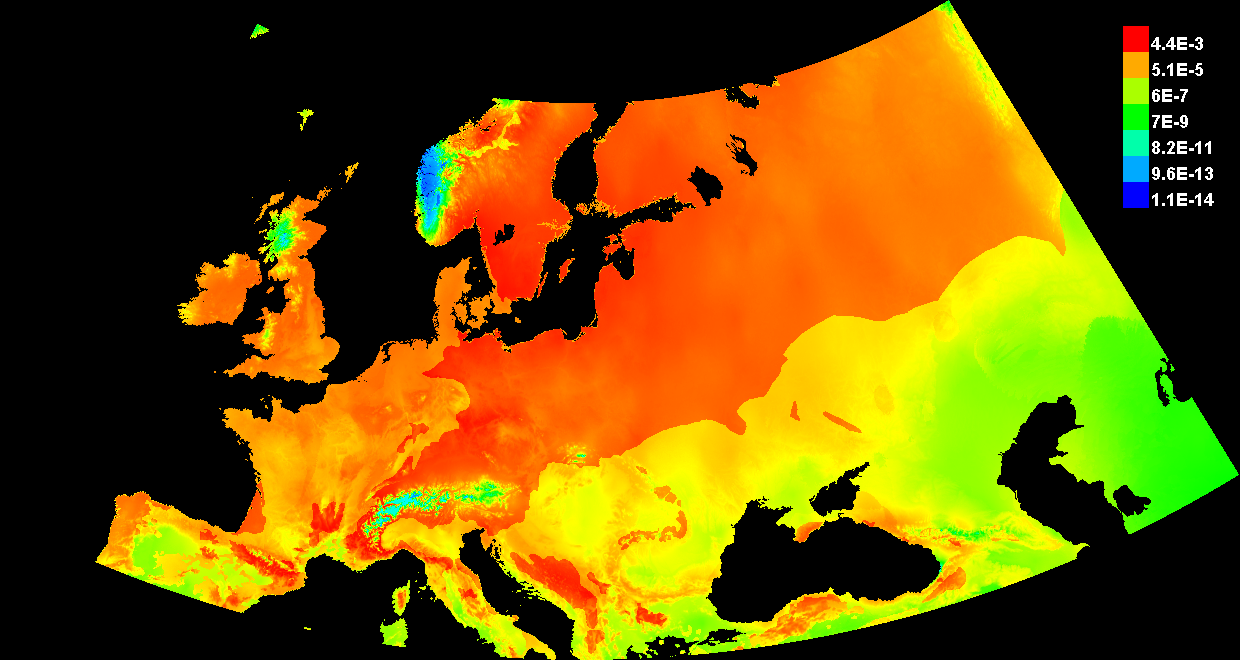

Supplement: Multimedia component 8 [file mmc8.zip › MaxEnt/plots/Aureoboletus_projectellus_PCA_lowerci.png]

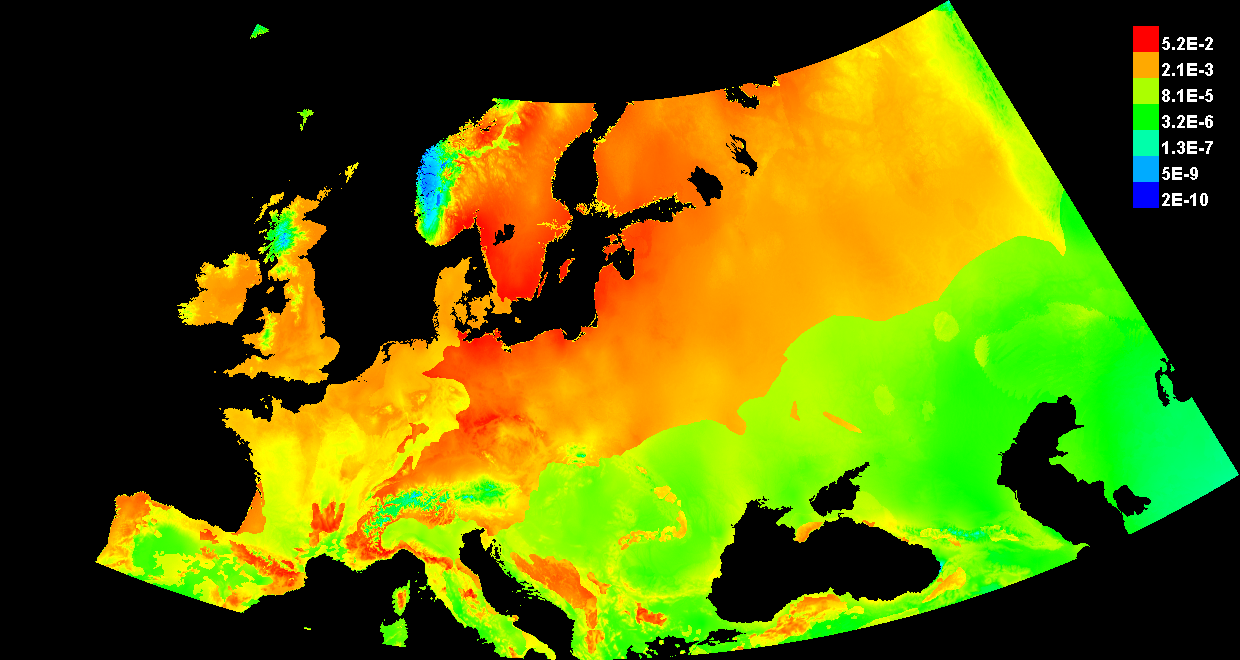

Supplement: Multimedia component 8 [file mmc8.zip › MaxEnt/plots/Aureoboletus_projectellus_PCA_max.png]

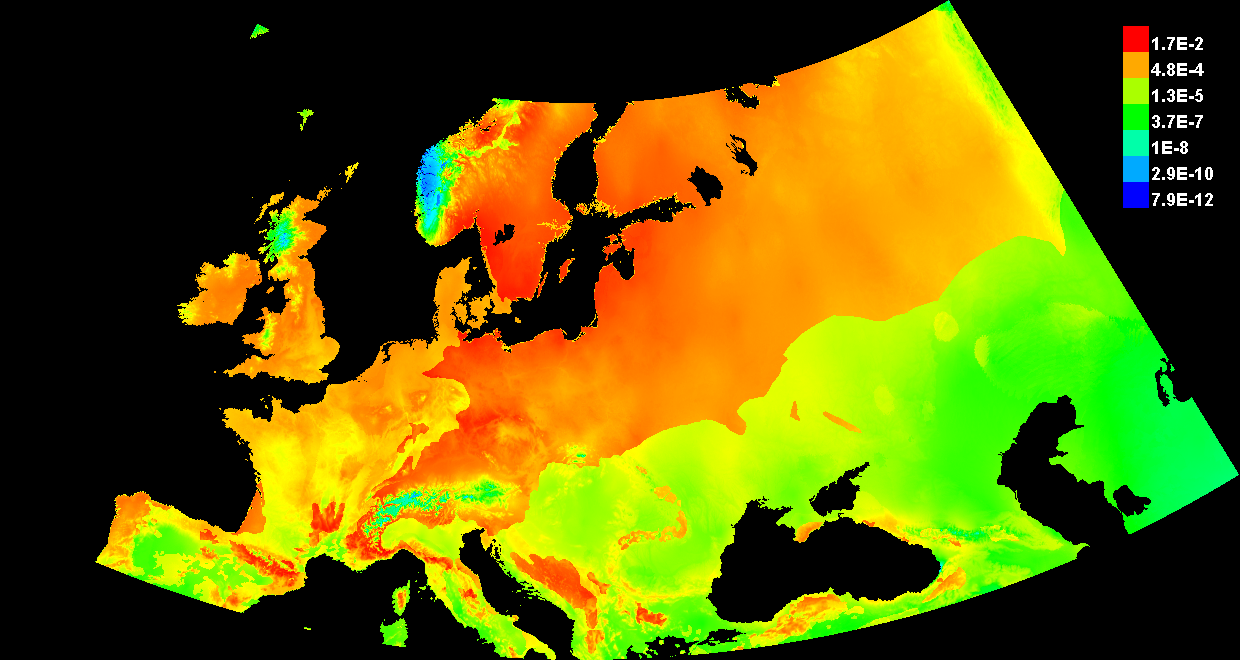

Supplement: Multimedia component 8 [file mmc8.zip › MaxEnt/plots/Aureoboletus_projectellus_PCA_median.png]

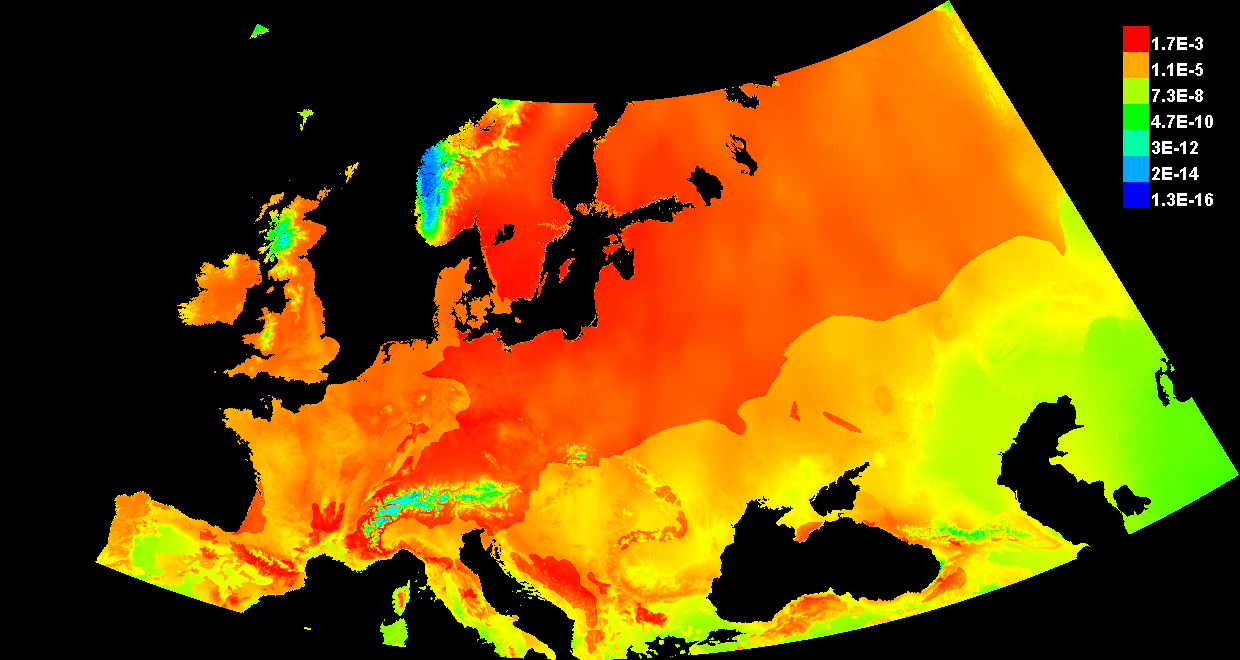

Supplement: Multimedia component 8 [file mmc8.zip › MaxEnt/plots/Aureoboletus_projectellus_PCA_min.png]

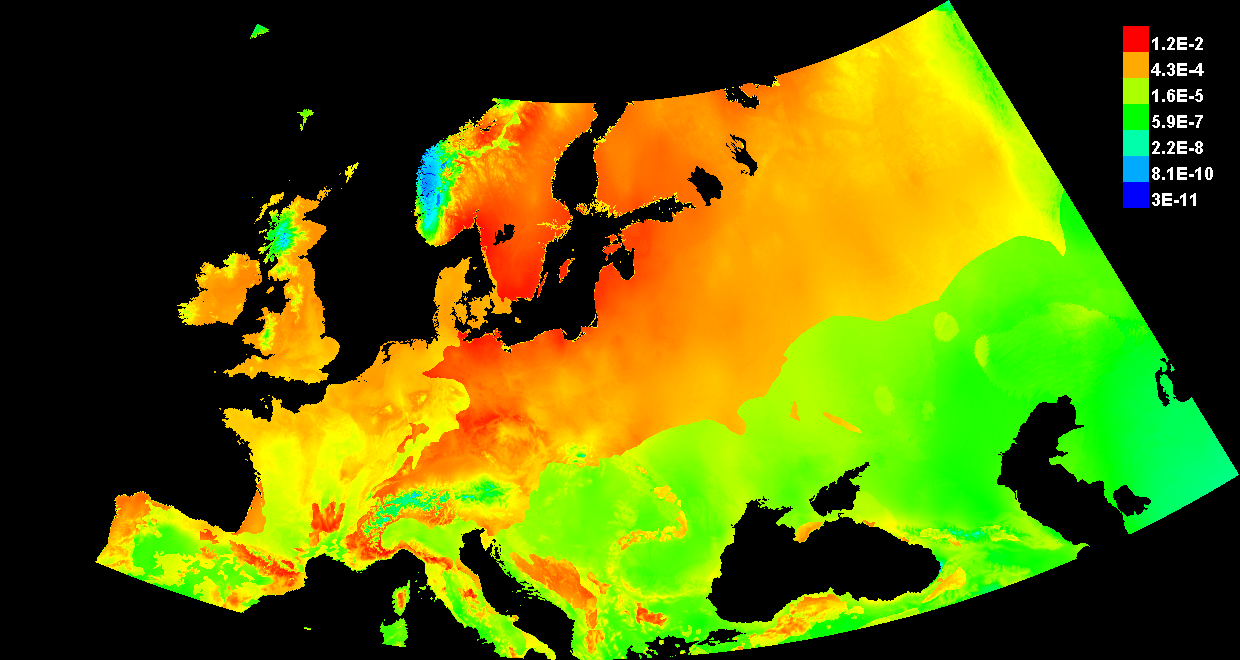

Supplement: Multimedia component 8 [file mmc8.zip › MaxEnt/plots/Aureoboletus_projectellus_PCA_stddev.png]

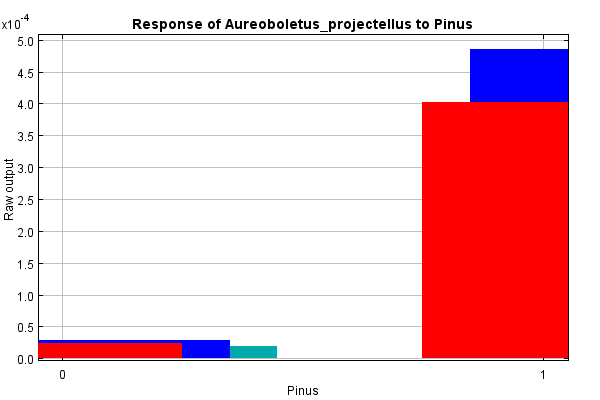

Supplement: Multimedia component 8 [file mmc8.zip › MaxEnt/plots/Aureoboletus_projectellus_Pinus.png]

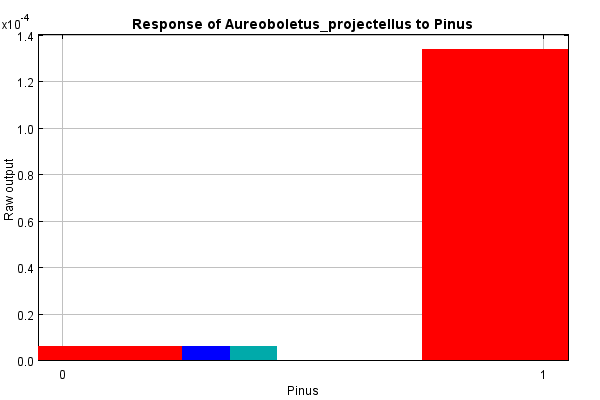

Supplement: Multimedia component 8 [file mmc8.zip › MaxEnt/plots/Aureoboletus_projectellus_Pinus_only.png]

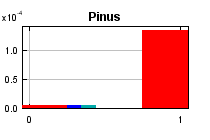

Supplement: Multimedia component 8 [file mmc8.zip › MaxEnt/plots/Aureoboletus_projectellus_Pinus_only_thumb.png]

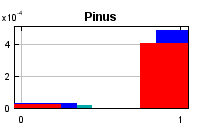

Supplement: Multimedia component 8 [file mmc8.zip › MaxEnt/plots/Aureoboletus_projectellus_Pinus_thumb.png]

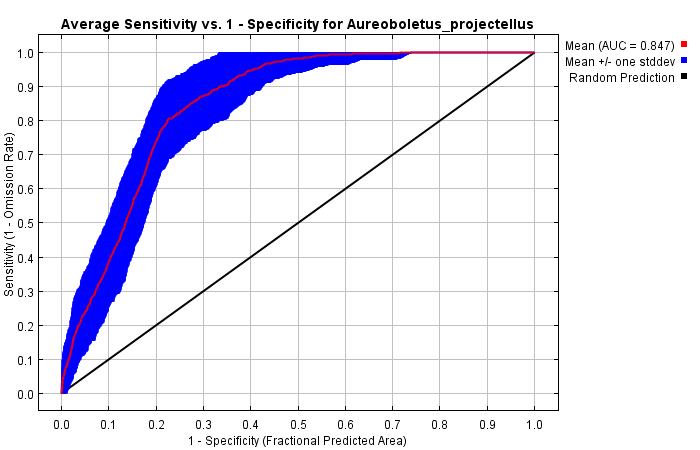

Supplement: Multimedia component 8 [file mmc8.zip › MaxEnt/plots/Aureoboletus_projectellus_roc.png]

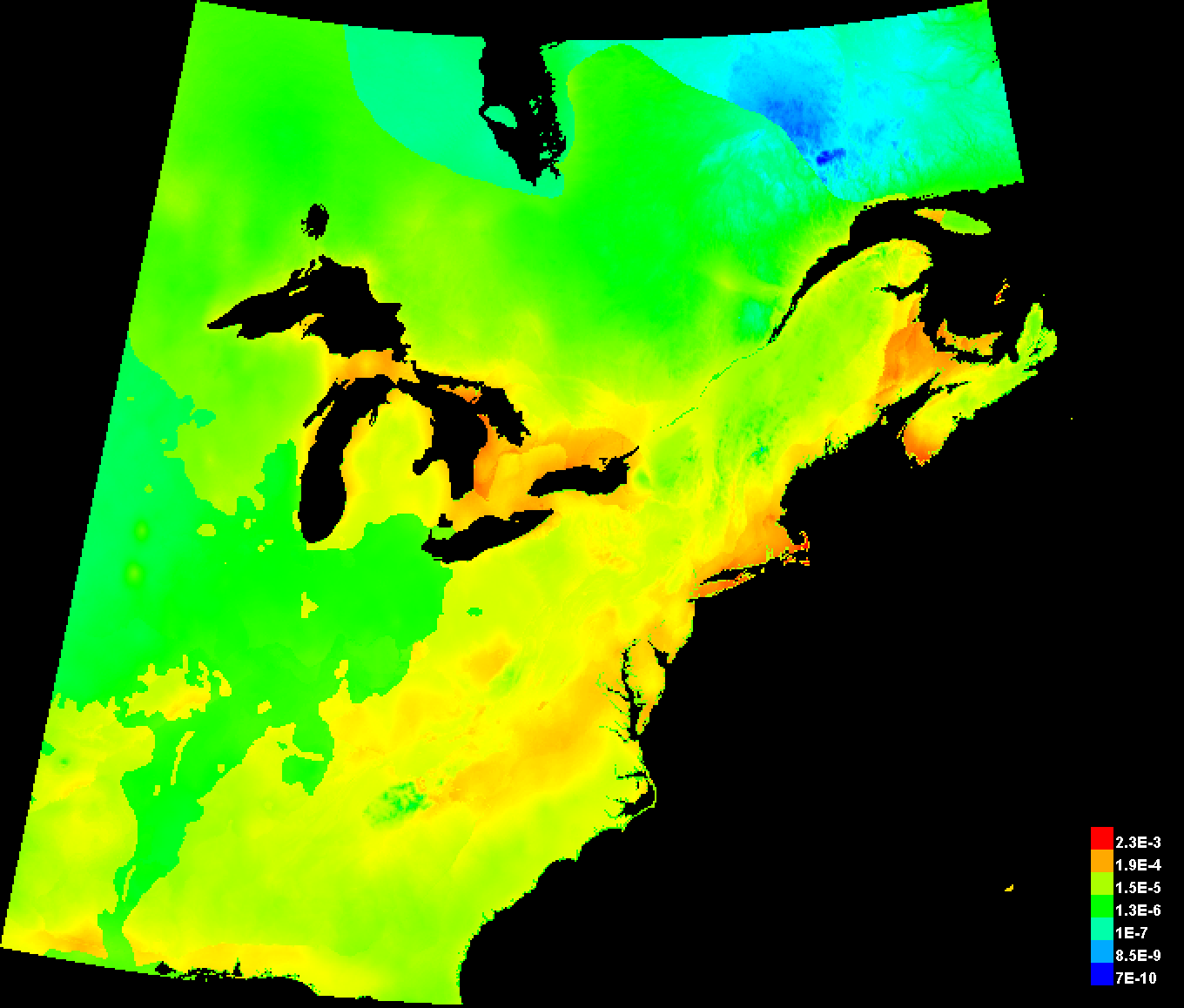

Supplement: Multimedia component 8 [file mmc8.zip › MaxEnt/plots/Aureoboletus_projectellus_stddev.png]
